# High concentrations of flavor chemicals are present in electronic cigarette refill fluids

**DOI:** 10.1038/s41598-019-39550-2

**Published:** 2019-02-21

**Authors:** Esther E. Omaiye, Kevin J. McWhirter, Wentai Luo, Peyton A. Tierney, James F. Pankow, Prue Talbot

**Affiliations:** 1 Environmental Toxicology Graduate Program, University of California, Riverside, CA 92521 United States; 2 Department of Molecular, Cell and Systems Biology, University of California, Riverside, CA 92521 United States; 3 Department of Civil and of Environmental Engineering, Portland State University, PO Box 751, Portland, OR 97207-0751 United States

## Abstract

We characterized the flavor chemicals in a broad sample of commercially available electronic cigarette (EC) refill fluids that were purchased in four different countries. Flavor chemicals in 277 refill fluids were identified and quantified by gas chromatography-mass spectrometry, and two commonly used flavor chemicals were tested for cytotoxicity with the MTT assay using human lung fibroblasts and epithelial cells. About 85% of the refill fluids had total flavor concentrations >1 mg/ml, and 37% were >10 mg/ml (1% by weight). Of the 155 flavor chemicals identified in the 277 refill fluids, 50 were present at ≥1 mg/ml in at least one sample and 11 were ≥10 mg/ml in 54 of the refill fluids. Sixty-one% (170 out of 277) of the samples contained nicotine, and of these, 56% had a total flavor chemical/nicotine ratio >2. Four chemicals were present in 50% (menthol, triacetin, and cinnamaldehyde) to 80% (ethyl maltol) of the samples. Some products had concentrations of menthol (“Menthol Arctic”) and ethyl maltol (“No. 64”) that were 30 times (menthol) and 100 times (ethyl maltol) their cytotoxic concentration. One refill fluid contained cinnamaldehyde at ~34% (343 mg/ml), more than 100,000 times its cytotoxic level. High concentrations of some flavor chemicals in EC refill fluids are potentially harmful to users, and continued absence of any regulations regarding flavor chemicals in EC fluids will likely be detrimental to human health.

## Introduction

Electronic cigarette (EC) consumers inhale aerosols that usually contain nicotine, propylene glycol and/or glycerol, and blends of flavor chemicals that directly contact the lining of the mouth and respiratory system^1,2^. Thousands of refill fluids, which are used at full strength, are commercially available for refilling cartomizer and tank-style EC products^3^. Instances of adverse health effects, some of which involve the respiratory system, such as bronchiolitis obliterans and acute eosinophilic pneumonia, have been attributed to EC use^4,5^. Cultured cells and animal models exposed to EC fluids and aerosols show increased oxidative stress, inflammatory responses, and impaired pulmonary defenses that may contribute to adverse health effects^6–9^.

The constituents of EC fluids and aerosols that cause adverse effects in cells and animals are beginning to be identified. Cytotoxicity of ECs has been linked to the presence of multiple flavor chemicals, including cinnamaldehyde^10–13^. As recently pointed out by the Flavor and Extracts Manufacturers’ Association (FEMA), while many of the flavor chemicals used in EC refill fluids are on the FEMA GRAS (generally regarded as safe) list, the GRAS designation presumes ingestion and does not apply to inhalation^14,15^. In addition, government agencies, such as the National Institute of Occupational Safety Health (NIOSH), have published inhalation exposure guidelines to protect workers who manufacture flavor chemicals from adverse health effects^16^. Clearly more data are needed to inform regulatory agencies and protect public health.

The purpose of this study was to identify and quantify the flavor chemicals in a broad spectrum (277) of EC refill fluids that were purchased in four countries to gain a better understanding of the range of chemicals and concentrations used in these products. Each flavor chemical was also classified based on organoleptic characteristics and their frequency of use in refill fluids. Two commonly used flavor chemicals were further evaluated for cytotoxicity using an *in vitro* model based on human respiratory cells.

## Results

### Identification and quantification of flavor chemicals by gas chromatography-mass spectrometry

**Figure 1:**
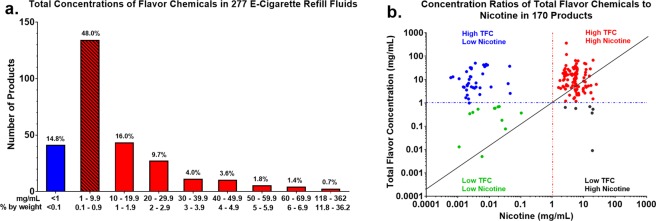
Total Concentrations of Flavor Chemicals and Nicotine in EC Refill Fluids. (**a**) The total concentration of flavor chemicals ranged from <1 mg/ml to 362 mg/ml. Total weight concentration of the flavor chemicals (mg/ml) was determined for each product and plotted according to the ranges in the figure. The numbers above the frequency bars represent the percentage of products in each group. (**b**) The concentration of nicotine (*x*-axis) plotted against the total concentration of flavor chemicals (*y*-axis) for each product, which ranged from 0.005–362 mg/ml.

Using authentic chemical materials purchased from chemical supply houses, analytical standards were prepared for 178 “target analytes”, namely 177 known flavor chemicals (including triacetin) plus nicotine. One hundred and fifty-five flavor chemicals in over 22 organoleptic groups were identified in our sample of 277 refill fluids (Supplemental Table 1). The sum of the detected flavor chemical concentration values in the 277 products ranged from a low of 0.005 mg/ml to a high of 362 mg/ml (Supplemental Table 2). About 85% (236 of 277) of the samples had total flavor chemical concentrations in excess of 1 mg/ml (Fig. 1a), in good agreement with a smaller sample set analyzed previously^17^, and about 37% (102 of 277) were >10 mg/ml. The detected concentrations of individual flavor chemicals ranged from 0.00085 to 343 mg/ml. Fifty chemicals were found in some samples at concentrations between 1–9.9 mg/ml, and 11 were found in some samples at concentrations ≥10 mg/ml (Supplemental Table 1). About 2.5% (7 of 277) of the samples had total flavor chemical concentrations less than 0.1 mg/ml. The brand/manufacture and product names of all 277 EC refill fluids evaluated are presented in Supplemental Table 3.

The 177 flavor chemicals on the target analyte list could not include every flavor chemical in the 277 products that were analyzed. The propylene glycol and glycerol acetals of cinnamaldehyde, vanillin, and ethyl vanillin were frequently detected in the refill fluids containing substantial cinnamaldehyde, vanillin, and ethyl vanillin. For the seven products with concentrations of total target flavor chemical values of <0.1 mg/ml, only small amounts of 2-hexanal and a few other non-target flavor chemicals were detected, indicating they were truly low/non-flavored fluids.

### Relationship of the total concentration of flavor chemicals to nicotine concentration

The total concentration of the flavor chemicals is plotted vs. nicotine concentration for the 170 refill fluids that contained nicotine in Fig. 1b. Detected nicotine concentrations ranged from <0.0006 mg/ml to 25.4 mg/ml. 116 out of the 170 products had nicotine concentrations ≥1 mg/ml (Fig. 1b), while 54 had concentrations <1 mg/ml (Fig. 1b). The nicotine and flavor chemicals that were <1 mg/ml may have been incidental, caused by carryover during manufacturing, or picked up during storage. For those products that contained nicotine >1 mg/ml, the ratio for total flavor chemicals/nicotine was greater than 2 for 56% of the samples, and for one product (“Cinnamon Bomb”), the ratio was 129. In Fig. 1b, points lying above the diagonal line have a total flavor concentration/nicotine concentration ratio greater than 2. The data demonstrate that flavor chemicals are major ingredients of many EC refill fluids, and often present at total concentrations higher than that of nicotine.

### Organoleptic properties and concentration ranges of 155 detected target analyte flavor chemicals

**Figure 2:**
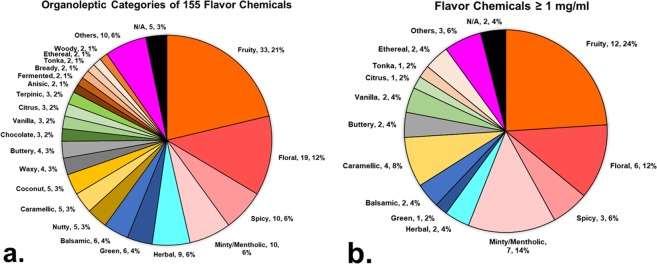
Organoleptic Properties of Flavor Chemicals Identified. (**a**) The taste and odor descriptions of flavor chemicals were obtained from an online database and charted to show the number and percentages of flavor chemicals in each category. The pie chart shows the top 4 tastes as fruity, floral, spicy and minty/mentholic. Nine chemicals grouped as “N/A” did not have any identified taste/description. (**b**) Flavor chemicals present in concentrations >1 mg/ml were then sorted to identify major and frequently used flavor categories.

The 155 target analyte flavor chemicals detected in the samples were grouped into flavor categories using reported taste and odor descriptions (aka “organoleptic properties”) (http://www.thegoodscentscompany.com/)^18^, (Supplemental Table 1 and Fig. 2a). The top five categories were “fruity” (21%), “floral” (12%), “spiced” (6%), “minty/menthol” (6%), and “herbal” (6%). “Popcorn”, “musty”, “phenolic”, “campherous”, “honey”, “meaty”, “smoky”, “tropical”, “earthy” and “odorless” flavor chemicals appeared only once and are grouped as “others”. Organoleptic information was not available (N/A) for strawberry glycidate_A, strawberry glycidate_B, heliotropin PG acetal, 4-methylbenzyl alcohol, and aromadendrene.

We further evaluated the organoleptic distribution of those chemicals that were present at concentrations greater than 1 mg/ml (Fig. 2b). The top categories in this analysis were “fruity”, “minty/mentholic”, “floral”, “caramellic”, and “spicy”. In the “others” category, acetylpyrazine (popcorn), hemineurine (meaty) and syringol (smoky) were also present at concentrations greater than 1 mg/ml.

### Frequency distribution, chemical class, and hazard classification of the 155 detected target analyte flavor chemicals

**Figure 3:**
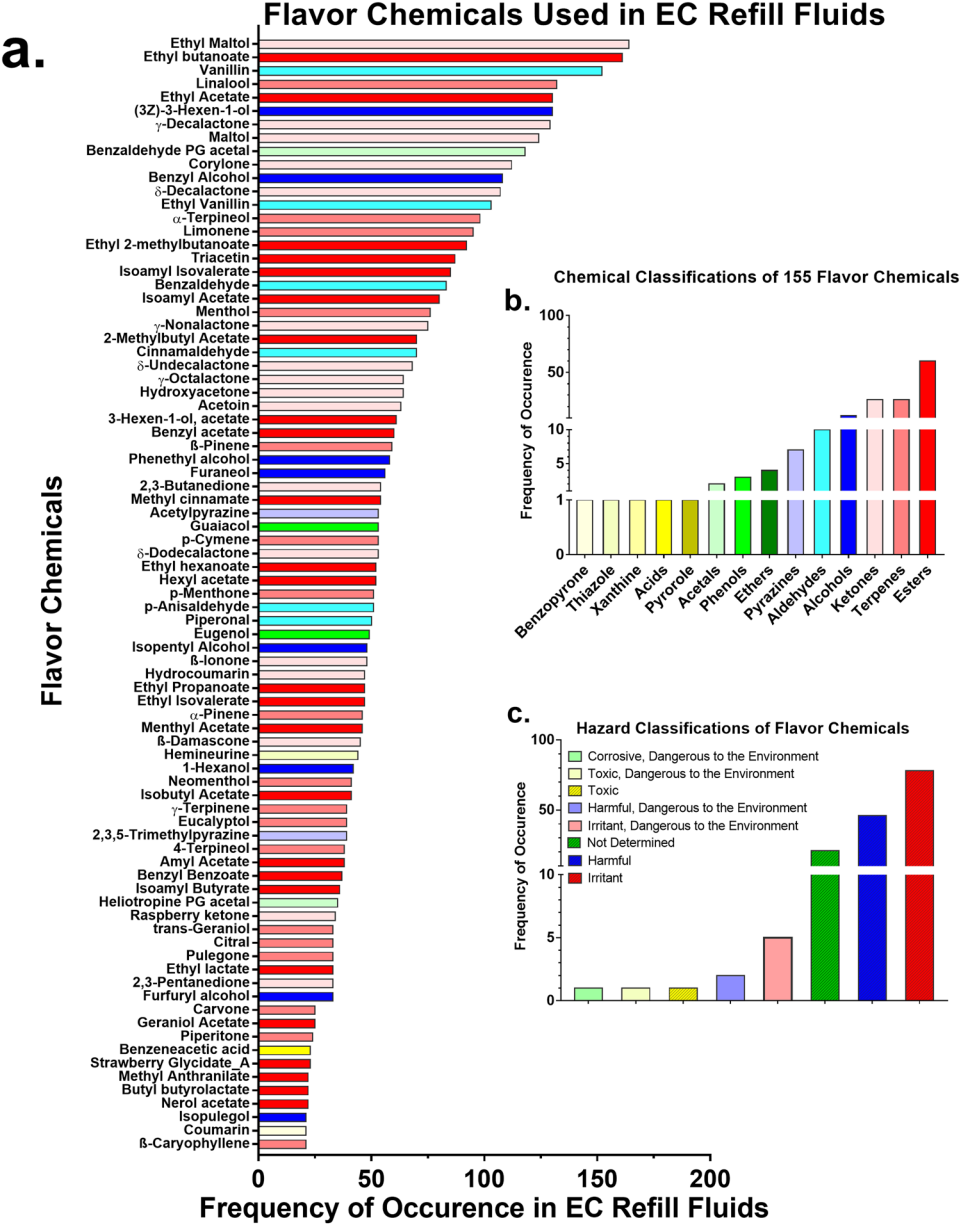
Frequency Distribution, Chemical Classes, and Hazard Classification of the Flavor Chemicals. (**a**) The frequency with which individual flavor chemicals were found in at least 21 products. The *x*-axis is the number of refill fluids in which the chemicals were found and the *y*-axis is sorted according to decreasing frequency of their occurrence. Frequency ranged from 21–164 with the highest being ethyl maltol. Chemicals appearing less frequently are shown in Supplemental Fig. 1. (**b**) The chemical classes of the flavor chemicals (*x*-axis) are plotted versus the frequency of occurrence of each class of flavor chemicals (*y*-axis). (**c**) The classification of flavor chemicals into color coded hazard categories using the European safety data (x-axis) are plotted versus the frequency of occurrence of flavor chemicals in each hazard category (*y*-axis).

The frequency with which each of the 155 detected target analyte flavor chemicals appeared in refill fluids is shown in Supplemental Table 1, Fig. 3a and Supplemental Fig. 1. The chemicals in Fig. 3a appeared in at least 21 different products out of 277 total. The 13 most frequently used flavor chemicals that appeared over 100 times in descending order of frequency were: ethyl maltol, ethyl butanoate, vanillin, linalool, ethyl acetate, (3z)-3-hexen-1-ol, γ-decalactone, maltol, benzaldehyde PG acetal, corylone, benzyl alcohol, δ-decalactone, and ethyl vanillin (Fig. 3a). The chemicals in Supplemental Fig. 1 appeared in 20 or fewer products.

Flavor chemicals were grouped into chemical classes using their structural properties (Fig. 3b). We used parent compound structures to classify those flavor chemicals that could be placed in more than one chemical group. About 39% (60 of 155) were esters followed by terpenes and ketones, which were both 16%. One flavor chemical each was classified as a pyrrole, acid, xanthine, thiazole and benzopyrone.

**Figure 4:**
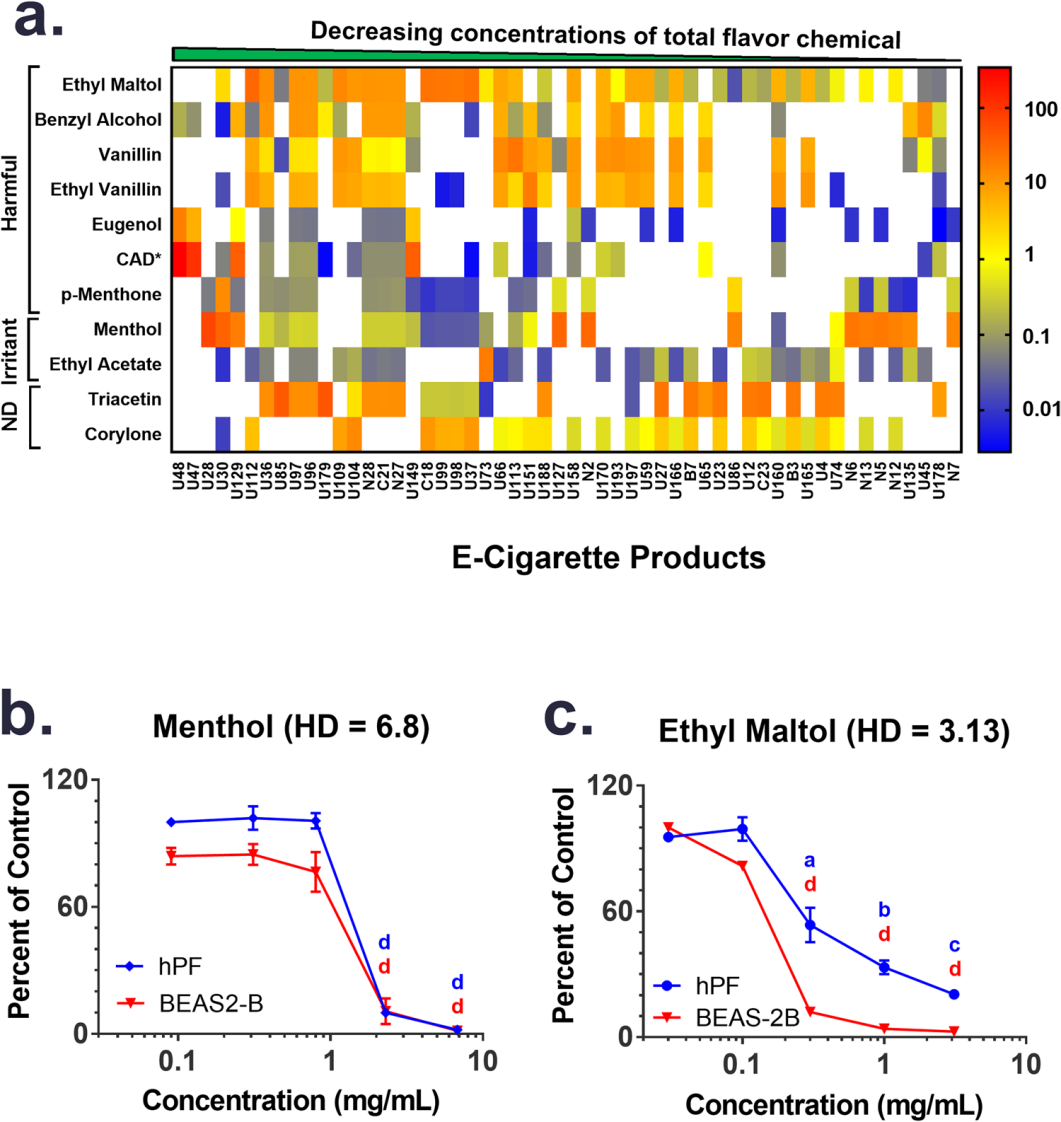
Heat Map and Cytotoxicity of Flavor Chemicals >10 mg/ml. (**a**) The *x*-axis of the heat map shows individual refill fluid products with at least one flavor chemical >10 mg/ml. Total flavor concentration decreases from left to right. The *y*-axis is ordered from high to low toxicity for the individual flavor chemicals based on the LD_50_ oral dose for rats (from peer reviewed articles on the Good Scents database (www.thegoodscentscompany.com) and grouped according to the European CLP regulation criteria; harmful; irritant, and not determined (ND). Concentration of individual flavor chemicals >1 mg/ml are shown as yellow cells and >10 mg/ml are orange to red cells. The country of each product’s origin is designated on the *x*-axis labels by U = USA, N = Nigeria, C = China, and B = Britain. Cinnamaldehyde is abbreviated CAD*. Dose-response curves for menthol (**b**) and ethyl maltol (**c**) tested with hPF and BEAS-2B cells in the MTT assay. The highest concentration of each flavor chemical tested is 10% of that found in the refill fluid. Each point is the mean ± standard error of the mean of three independent experiments. Points with letters are significantly different from the untreated control and points with different letters show degrees of statistical significance. ^a^p < 0.05, ^b^p < 0.01, ^c^p < 0.001, ^d^p < 0001.

Using available safety information^18^, all the flavor chemicals were grouped in terms of potential to cause harm (Fig. 3c). This hazard classification is based on: (1) the Dangerous Substances Directive^19^ for pure substances; and, (2) the Dangerous Preparations Directive^20^ for mixtures. Some provisions of both directives related to classification, packaging and labeling of dangerous substances and preparations were amended and replaced by the Regulation on the Classification, Labeling and Packaging (CLP) of Substances and Mixtures^21^, which was enacted in 2008 with enforcement beginning in 2009. According to these directives the categories applicable to the flavor chemicals in our study included; (1) “irritants”, (2) “harmful”, (3) “toxic/harmful and dangerous to the environment”, and (4) not determined (Figs. 3c and 4). Most of the chemicals were “irritants” and “harmful”, and three (limonene, strawberry glycidate_A and strawberry glycidate_B) were both “irritants” and “dangerous to the environment”. One chemical, allyl hexanoate, was “toxic and dangerous to the environment”. Irritants are chemicals that can potentially destroy living tissues at significant doses. Whether or not any of these chemicals would adversely affect EC users would depend on their concentration, extent of consumption, and sensitivity of the user.

### Flavor chemicals >10 mg/ml in EC refill fluids

A heat map was created to visualize the concentrations and frequency of use of the 11 chemicals that were present in at least one product at a concentration >10 mg/ml (Fig. 4a). The heat map shows: (1) 11 chemicals with individual concentrations >10 mg/ml in at least one refill fluid, (2) the relative frequency with which they were found, and (3) their concentrations in each product. Some chemicals appeared frequently at concentrations >10 mg/ml (e.g., ethyl maltol and ethyl vanillin), while others appeared at >10 mg/ml in only one product (e.g., ethyl acetate and p-menthone).

Data on the inhalation toxicity of flavor chemicals are scarce, therefore we ranked these chemicals on the *y*-axis (most to least toxic) based on previously published peer reviewed oral toxicity data in rats (Fig. 4a)^18^. Nine were categorized as harmful or irritants. Four of these chemicals were present in 50% (menthol, triacetin, and cinnamaldehyde) and 80% (ethyl maltol) of the samples. Two of these flavor chemicals had no available oral toxicity data (ND). One product, which was compounded in a local vape shop and sold as a refill fluid, had 343 mg/ml (~34%) of cinnamaldehyde, which is more than 100,000 times the cytotoxic level we reported previously^10,12^.

### Cytotoxicity of menthol and ethyl maltol

Because ethyl maltol was in almost all products, often at concentrations >1 mg/ml, and because menthol was highest in concentration (after cinnamaldehyde which was previously tested), authentic standards of each were evaluated for cytotoxicity using the MTT assay with human pulmonary fibroblasts (hPF) and human lung epithelial cells (BEAS-2B). The results are summarized in Fig. 4b and c, for which the highest concentration on the *x*-axis is only 10% of the concentration found in at least one of the refill fluids. Both flavor chemicals were highly cytotoxic at concentrations 30 (menthol) and 100 times (ethyl maltol) lower than the highest concentrations in the refill fluids. BEAS-2B cells (IC_50_ = 0.15) were somewhat more sensitive to ethyl maltol than hPF (IC_50_ = 0.28).

## Discussion

EC manufacturers have about 16,000 flavor chemicals from which to choose^15^. Our data provide a simpler picture: (1) the number that were used in our sample of 277 refill fluids was 155, not thousands; (2) in any given product, the number of flavor chemicals typically ranged from 0 to 50; and (3) while some constituents were present at rather low concentrations, 11 were found at concentrations >10 mg/ml. When evaluating just those chemicals that were over 1 mg/ml, the number per product ranged from 0 to 10. Moreover, the total concentrations of flavor chemicals exceeded the nicotine concentration in over half of the products. These data demonstrate that flavor chemicals are a major component of currently marketed EC refill fluids and their health effects on EC users should be addressed.

Of particular importance in our study is the finding that some products have individual flavor chemicals in concentrations >10 mg/ml, and many of these chemicals were found in many of the samples (e.g., ethyl maltol was in 24.5% of the products at ≥10 mg/ml, and menthol was in 22.6% of the products at ≥10 mg/ml) (Fig. 4). Based on the results of the MTT assay, menthol and ethyl maltol were present at concentrations that would be cytotoxic in 34% (26 of 76) and 40% (66 of 164) of the refill fluids that contained menthol and ethyl maltol, respectively. While the MTT data cannot be translated directly to *in vivo* human effects, they do raise concern about the potential for these chemicals to cause harm to users at the concentrations currently used in some refill fluids. Moreover, chronic exposure to high concentrations of flavor chemicals may be far more damaging than the effects seen in our acute experiments.

Further evidence that the concentrations of some flavor chemicals used in EC refill fluids may exceed safe levels can be found by comparing our data to the concentrations in other consumer products. Although cinnamaldehyde has been approved by the Food and Drug Administration (21CFR182.60) for use as a flavoring agent^22^ and given FEMA GRAS status, some in the flavor industry and the Research Institute for Fragrance Materials have recommended that cinnamaldehyde not exceed 1% when used in skin cosmetic products^23,24^. Cinnamaldehyde is usually found in body care and household products, such as detergents, creams and lotions, soaps and perfumes, in the range 0.001–0.8%^25^. Moreover, cinnamaldehyde is used in food products at concentrations ranging from 7.7 ppm (0.00077%) in ice creams to a 700 ppm (0.07%) in candy and up to a 6,400 ppm (0.64%) in fruits and juices^23,26,27^. In our refill fluid samples, two products had cinnamaldehyde concentrations of 118 mg/ml (11.8% or 118,000 ppm) and 343 mg/ml (34.3% or 343,000 ppm). We have previously reported that the cinnamaldehyde concentrations in a different set of refill fluid samples often exceeded 1% (range = 0.00022–14%) for cinnamon flavored refill fluids^10,11^. Our current study further shows, in agreement with our earlier work^11^ that cinnamaldehyde is more widely used in EC refill fluids than would be expected based on the names of the EC products. For example, cinnamaldehyde was found previously in fruity flavors, such as a product named “Blueberry Hills”, and in the current study was found in 70 of 277 (25%) products, even though only two products indicated “cinnamon” in their name. Cinnamaldehyde at concentrations found in EC products has also been shown to impair the function of immune cells in the respiratory system^13^.

Like cinnamaldehyde, ethyl maltol is added to edible products such as beverages, ice cream, candy, baked goods, gelatin desserts, meat, chewing gum and related products in concentrations up to 0.0142%^28^, and the maximum concentrations of ethyl maltol in final formulations of soap, detergents, and creams and lotions are 0.06%, 0.006%, and 0.01%, respectively^29^. These concentrations of ethyl maltol in consumer products are far below the concentrations that we found (0.008–3.13%) in 46% of the of the refill fluids that we tested. Ethyl maltol increases free radical formation in EC aerosols^30^, which could further increase the toxicity of products with this flavor chemical.

Menthol is commonly used in consumer products including tobacco cigarettes. Mentholated cigarettes generally have menthol concentrations <7 mg/cigarette and many are <0.002 mg/cigarette^31^. Menthol was present in 76 of our refill fluids at concentrations ranging from 0.002 to 68 mg/ml. Twelve out of the 76 refill fluids had concentrations greater than 10 mg/ml, which would exceed the concentrations normally found in conventional tobacco cigarettes flavored with menthol. Menthol produced cytotoxicity in the MTT assay at concentrations 30 times lower than the highest concentration found in the refill fluids we analyzed.

2,3-butanedione (diacetyl), which can cause bronchiolitis obliterans, also called “popcorn lung disease”^32–35^, has previously been found in EC products^36,37^. We found diacetyl, as well as two related chemicals, acetoin and 2,3-pentanedione, in 54% of the refill fluids. Of these chemicals, diacetyl, acetoin and 2,3-pentanedione were present in 36% (54 of 150), 42% (63 of 150) and 22% (33 of 150), respectively. Assuming a consumer vapes 3.4 ml of a refill fluid^38^ containing diacetyl at 0.32 mg/ml (highest concentration found in our study) and the transfer rate of diacetyl to the aerosol is 100%, the consumer would be exposed to 1.088 mg of diacetyl/day (equivalent to 85.83 ppb/8 hour average) which is well above the exposure limit of 5 ppb for 8 hours recommended by NIOSH^39^. Concentrations in refill fluids also exceeded the Short-Term (15 minute) Exposure Limit of 25 ppb for diacetyl^39^. These data raise concern about the potential for harm of some of the flavor chemicals that are present in refill fluids at relatively low concentrations.

Coumarin (1,2-benzopyrone) is another chemical of concern. It was present in 21 products at concentrations ranging from 0.007 to 5 mg/ml. Coumarin is currently prohibited as an additive in human food by the Food and Drug Administration (21CFR189.130) due to its hepatotoxicity, and when present, the food is deemed adulterated^40^. It’s prohibition in food supports the idea that it should likewise not be used in tobacco products, including ECs. Coumarin is often co-extracted from cinnamon with cinnamaldehyde and may have been a co-constituent inadvertently introduced into the products containing high concentrations of cinnamaldehyde

Our data show that both menthol and ethyl maltol are frequently used in refill fluids at concentrations that were cytotoxic to cultured human lung cells when tested with the MTT assay. Menthol and ethyl maltol have been reported in other brands of EC products^41–43^, although their concentrations were not given. While most prior work on the toxicity of EC flavors has been done on intact fluids^9,44–46^, several studies have examined the cytotoxicity of authentic standards of flavor chemicals present in EC fluids and aerosols^41,47,48^.

Our cytotoxicity data with menthol and ethyl maltol can be compared to results reported previously. Both ethyl maltol and menthol altered calcium homeostasis in CALU3 lung epithelial cells by depleting the endoplasmic reticulum of Ca^2+^ and elevating cytosolic Ca^2+^ ^41^. The effective concentration (EC_50_) of menthol in the Ca^2+^ assay (3.02 mM)^41^ was similar to the inhibitory concentration (IC_50_) of menthol in our MTT assay (1.38 mg/ml or 8.8 mM). In contrast, the concentration of ethyl maltol (0.15 mg/ml or 1.07 mM) that produced an effect in our MTT assay was much lower than the effective concentration (21.14 mM) in the Ca^2+^ influx assay^41^. These differences with ethyl maltol could be related to the different cell types (BEAS-2B versus CALU3) that were used in the two studies. These data show that mitochondrial reductase activity (MTT assay) is very sensitive to ethyl maltol and demonstrate the importance of evaluating multiple toxicity endpoints.

Cinnamaldehyde, which was very high in concentration in several products in the current study, was shown previously to be highly cytotoxic and immunosuppressive when tested *in vitro* with lung cells^10,11,13,48^. Based on our prior data with the MTT assay^10,11^, cinnamaldehyde is the most potent flavor chemicals we have tested, and it was found in 25% of all refill fluids in the current study.

Aerosolization of flavor chemicals can increase aldehyde concentrations in EC aerosols^49^, although this was not confirmed in a second study^50^. A previous study which compared the toxicity of EC aerosol produced at 3 versus 5 volts (4.3 W versus 11.9 W) showed a clear increase in toxicity at the higher voltage^48^. This observation would be consistent with the production of toxic reaction products upon aerosolization at the higher voltage and deserves further evaluation given the high concentration of flavor chemicals that we report here in many refill fluids, and the increased popularity of tank style EC with variable power controls.

## Conclusions

This paper is the first to identify and quantify the flavor chemicals in a broad spectrum of EC refill fluids that are sold worldwide. These data should help focus future work on the flavor chemicals that are frequently used and/or used at high concentrations. Our findings draw attention to the fact that ECs serve the dual purpose of delivering both nicotine and flavor chemicals and that some of the flavor chemicals are used at concentrations far in excess of the acceptable levels found in other consumer products. The human health effects of inhalation of flavor chemicals at high concentrations are not well understood and will require further evaluation with attention to those chemicals that are frequently used in high concentration and cytotoxic *in vitro*.

There are now sufficient data to heighten concern about the unregulated use of flavor chemicals in refill fluids, especially at high concentrations. Given the current data, regulation of flavor chemicals in EC products should be addressed, as we have recommended previously^17^. Regulatory agencies could consider limiting the concentrations of flavor chemicals in EC products, requiring a list of flavor ingredients on product labels, restricting use of flavor chemicals that are cytotoxic at low concentrations, such as cinnamaldehyde, or banning the use of flavor chemicals in tobacco products, as suggested by others^51^.

## Materials and Methods

### Sampling

A worldwide sample of 277 bottles of EC refill fluids was purchased from product lines offered by manufacturers in the USA, England, China, and Nigeria, and seven fluids were compounded for us by a vape shop in Riverside, CA to match popular flavor names not offered by the shop. The latter group of seven products was included to begin an examination of what may result from fluid “cloning” services offered by some EC vendors. Flavor chemicals were analyzed by gas chromatography-mass spectrometry (GC-MS), and two chemicals found at high concentrations were tested for cytotoxicity using the MTT assay with BEAS-2B bronchial epithelial cells and human pulmonary fibroblasts (hPF), as described previously^10–12^.

### Identification and quantification of flavor chemicals in EC refill fluids

For each refill fluid, 50 µl were dissolved in 0.95 ml of isopropyl alcohol (IPA) (Fisher Scientific, Fair Lawn, NJ). All diluted samples were shipped overnight on ice to Portland State University and analyzed using GC-MS on the day they were received. A 20 µl aliquot of internal standard solution (2000 ng/µl of 1, 2, 3-trichlorobenzene dissolved in IPA) was added to each diluted sample before analysis. Using internal standard-based calibration procedures described elsewhere^52^, analyses were performed with an Agilent 5975 C GC-MS system (Santa Clara, CA). A Restek Rxi-624Sil MS column (Bellefonte, PA) was used (30 m long, 0.25 mm id, and 1.4 µm film thickness). A 1.0 µl aliquot of diluted sample was injected into the GC with a 10:1 split. The injector temperature was 235 °C. The GC temperature program for analyses was: 40 °C hold for 2 min; 10 °C/min to 100 °C; then 12 °C/min to 280 °C and hold for 8 min at 280 °C, then 10 °C/min to 230 °C. The MS was operated in electron impact ionization mode at 70 eV in positive ion mode. The ion source temperature was 220 °C and the quadrapole temperature was 150 °C. The scan range was 34 to 400 amu. Each of the 178 target analytes was quantitated using authentic standard material and an internal standard compound normalized multipoint calibration.

### Cell Culture

Human pulmonary fibroblasts (hPF) (ScienCell, Carlsbad, CA) were cultured in complete fibroblast medium supplemented with 2% fetal bovine serum, 1% fibroblast growth serum, and 1% penicillin/streptomycin prepared according to the manufacturer’s protocol^10,12^. Prior to culturing, Nunc T-25 tissue culture flasks (Fisher Scientific, Tustin CA) were coated with poly-L-lysine (PLL) prepared at a 20ul/10 ml concentration and kept in the incubator to allow for even distribution and efficient coating of the culture flask. hPF cultures were maintained in 5% CO_2_ at 37 °C and 95% relative humidity and the medium was replaced every 48 hours. At 80–90% confluency, cells were harvested using Dulbecco’s Phosphate Buffered Saline (DPBS) for washing and incubated with 0.01% trypsin-EDTA/DPBS (GIBCO, InVitrogen Carlsbad, CA) for 2 mins at 37 °C to allow detachment from the PLL coated surface of the culture flask. Detached cells were washed with culture medium and spun at 3,000 g for 3 mins. The resulting supernatant was discarded, and cell pellets were resuspended in fresh culture medium for the MTT cytotoxicity experiments. Single cells were plated at a density of 3,000 cells/well (cells/0.32 cm^2^) based on a standard curve produced using a BioMate 3S Spectrophotometer (Thermo Fisher Scientific, Chino, CA) and evenly dispersed in 96-well plates.

Human bronchial epithelial (BEAS-2B) cells were cultured in basal BEBM (Lonza, Walkersville, MD) supplemented with 2 ml bovine pituitary extract and 0.5 ml of insulin, hydrocortisone, retinoic acid, transferrin, triiodothyronine, epinephrine, and human recombinant epidermal growth factor (Lonza, Walkersville, MD). Nunc T-25 tissue culture flasks were coated overnight with BEBM, collagen, BSA and fibronectin prior to culturing and passaging cells. At 80% confluency, cells were harvested using DPBS for washing and incubated with 1.5 ml of 0.25% trypsin-EDTA (GIBCO, InVitrogen Carlsbad, CA) and poly-vinyl-pyrrolidone for 3–4 mins at 37 °C to allow detachment. Cells were cultured in T-25 flasks at 75,000 cells/flask, and the medium was replaced the next day and then every other day. Plating for the MTT assay was done at 3,500 cells/well in pre-coated 96-well plates.

### Cytotoxicity of authentic standards of flavors chemicals

Authentic standards of menthol and ethyl maltol (Sigma-Aldrich, St Louis, MO) were tested individually using the MTT assay with hPF and BEAS-2B cells. The MTT assay was performed over 3-fold dilutions with the highest concentration being 10% of the concentration found in the refill fluids. Concentrations above 10% were not used as they produced a vapor effect^9^ that shifted the dose response curve to the left. Serial dilutions of authentic standard solutions in culture medium were arranged in 96-well plates with two negative controls next to the highest dose to check for a vapor effect^9^. Cells were allowed to attach for 24 hours, then treated for 48 hours after which 20 µl of MTT (Sigma-Aldrich, St Louis, MO) dissolved in 5 mg/ml of DPBS (Fisher Scientific, Chino, CA) were added to each well and incubated for 2 hrs at 37 °C. Solutions were removed, and 100 µl of dimethyl sulfoxide (DMSO) (Fisher Scientific, Chino, CA) were added to each well and gently mixed on a shaker. The assay was performed in triplicate, and the absorbance of control and treated wells was read against a DMSO blank at 570 nm using an Epoch micro-plate reader (Biotek, Winooski, VT). Each chemical was tested in three independent experiments.

### Data analysis

For the GC/MS results, the sample-mean values were analyzed using Prism software (GraphPad, San Diego, CA). MTT data were normalized by setting treatment wells as percentages of the negative control (100%). Prism software (GraphPad, San Diego, CA) was used to compute IC_50_s using the log inhibitor vs. normalized response-variable slope with the top and bottom constraints set to <100% and >0%, respectively. Graphs were plotted using GraphPad Software. When significance was found using a one-way analysis of variance, each concentration was compared to the control using Dunnett’s post hoc test.

## Supplementary information

Supplemental File

## Data Availability

All data are available within the manuscript.
