# Supplementary material for: High concentrations of flavor chemicals are present in electronic cigarette refill fluids
Source: Sci Rep. 2019 Feb 21;9:2468. doi: 10.1038/s41598-019-39550-2 (PMC6385236; doi:10.1038/s41598-019-39550-2)
Supplement: Supplementary file 1 — Supplemental File [file 41598_2019_39550_MOESM1_ESM.pdf]

Supplemental Material

High concentrations of flavor chemicals are present in electronic cigarette refill fluids

Esther E. Omaiye, Kevin J. McWhirter, Wentai Luo, Peyton A. Tierney, James F. Pankow and Prue Talbot

Supplemental Table 1: Properties, Frequency and Concentration Ranges of 155 Flavor Chemicals

Organoleptic Properties	Chemical Name	Frequency	≥ 10 mg/ml		1 - 9.9 mg/ml		≤ 1 mg/ml	
			[a] #	[b] %	#	%	#	%
Fruity	*Triacetin*	*87*	*19*	*21.8*	*21*	*24.1*	*47*	*54.0*
	Ethyl butanoate	*161*			*14*	*8.7*	*147*	*91.3*
	Isoamyl acetate	*79*			*12*	*15.2*	*67*	*84.8*
	γ-Decalactone	*129*			*6*	*4.7*	*123*	*95.3*
	Hexyl acetate	*52*			*6*	*11.5*	*46*	*88.5*
	Ethyl lactate	*33*			*4*	*12.1*	*29*	*87.9*
	Ethyl propanoate	*47*			*2*	*4.3*	*45*	*95.7*
	Methyl anthranilate	*22*			*2*	*9.1*	*20*	*90.9*
	Allyl hexanoate	*18*			*2*	*11.1*	*16*	*88.9*
	Benzaldehyde	*83*			*1*	*1.2*	*82*	*98.8*
	2-Methylbutyl acetate	*70*			*1*	*1.4*	*69*	*98.6*
	Isobutyl acetate	*41*			*1*	*2.4*	*40*	*97.6*
	Amyl acetate	*38*					*38*	*100*
	Ethyl 2-methylbutanoate	*92*					*92*	*100*
	Isoamyl isovalerate	*85*					*85*	*100*
	δ-Undecalactone	*68*					*68*	*100*
	Ethyl hexanoate	*52*					*52*	*100*
	Ethyl isovalerate	*47*					*47*	*100*
	β-Damascone	*43*					*43*	*100*
	Isoamyl butyrate	*35*					*35*	*100*
	Raspberry ketone	*34*					*34*	*100*
	Benzyl butyrate	*17*					*17*	*100*
	2-Hexen-1-ol	*16*					*16*	*100*
	Amyl isovalerate	*16*					*16*	*100*
	Benzyl propionate	*10*					*10*	*100*
	Raspberry Ketone methyl ether	*7*					*7*	*100*
	Butyl butyrate	*6*					*6*	*100*
	p-Tolualdehyde	*5*					*5*	*100*
	Methyl 2-methylbutyrate	*3*					*3*	*100*
	Ethyl heptanoate	*3*					*3*	*100*
	Ethyl isobutyrate	*2*					*2*	*100*
	cis-Limonene oxide	*2*					*2*	*100*
	Amyl butyrate	*1*					*1*	*100*
Floral	*Benzyl alcohol*	*108*	*8*	*7.4*	*35*	*32.4*	*65*	*60.2*
	Benzaldehyde PG acetal	*118*			*7*	*5.9*	*111*	*94.1*
	Linalool	*132*			*1*	*0.8*	*131*	*99.2*
	Piperonal	*50*			*1*	*2.0*	*49*	*98.0*
	Geraniol acetate	*25*			*1*	*4.0*	*24*	*96.0*
	trans-Geraniol	*33*			*1*	*3.0*	*32*	*97.0*
	Benzyl acetate	*60*					*60*	*100*
	Phenethyl alcohol	*58*					*58*	*100*
	(E)-β-Ionone	*48*					*48*	*100*
	Benzeneacetic acid	*23*					*23*	*100*
	Nerol acetate	*33*					*33*	*100*
	α-Ionone	*16*					*16*	*100*
	Acetophenone	*15*					*15*	*100*
	Methyl N-methylanthranilate	*14*					*14*	*100*
	Ethyl anthranilate	*9*					*9*	*100*
	α-Damascone	*7*					*7*	*100*
	Linaly propionate	*5*					*5*	*100*
	Benzyl benzeneacetate	*4*					*4*	*100*

	trans-Linalool Oxide	*2*					*2*	*100*
Menthol/Minty	*Menthol*	*76*	*12*	*15.8*	*18*	*23.7*	*46*	*60.5*
	p-Menthone	*51*	*1*	*2.0*	*13*	*25.5*	*37*	*72.5*
	Carvone	*25*			*2*	*8.0*	*23*	*92.0*
	Neomenthol	*41*			*1*	*2.4*	*40*	*97.6*
	Menthyl acetate	*46*			*1*	*2.2*	*45*	*97.8*
	Pulegone	*33*			*1*	*3.0*	*32*	*97.0*
	Isopulegol	*21*			*1*	*4.8*	*20*	*95.2*
	Methyl salicylate	*19*					*19*	*100*
	Ethyl salicylate	*4*					*4*	*100*
	Ethyl benzoate	*3*					*3*	*100*
Spicy	*Cinnamaldehyde*	*70*	*4*	*5.7*	*2*	*2.9*	*64*	*91.4*
	Eugenol	*49*	*1*	*2.0*	*4*	*8.2*	*44*	*89.8*
	4-Terpineol	*38*			*1*	*2.6*	*37*	*97.4*
	β-Caryophyllene	*21*					*21*	*100*
	β-Myrcene	*19*					*19*	*100*
	Isoeugenol methyl ether	*12*					*12*	*100*
	o-methoxycinnamaldehyde	*8*					*8*	*100*
	Acetyleugenol	*7*					*7*	*100*
	Eugenol methyl ether	*4*					*4*	*100*
	Cinnamyl acetate	*1*					*1*	*100*
Caramellic	*Ethyl maltol*	*164*	*13*	*7.9*	*65*	*39.6*	*86*	*52.4*
	Corylone	*111*	*3*	*2.7*	*51*	*45.9*	*57*	*51.4*
	Maltol	*124*			*21*	*16.9*	*103*	*83.1*
	Furaneol	*56*			*20*	*35.7*	*36*	*64.3*
	Hydroxyacetone	*60*					*60*	*100*
Vanilla	*Vanillin*	*152*	*9*	*5.9*	*54*	*35.5*	*89*	*58.6*
	Ethyl vanillin	*103*	*4*	*3.9*	*45*	*43.7*	*54*	*52.4*
	Isosafroeugenol	*19*					*19*	*100*
Ethereal	*Ethyl acetate*	*130*	*1*	*0.8*	*5*	*3.8*	*124*	*95.4*
	Butyl acetate	*8*			*1*	*12.5*	*7*	*87.5*
Buttery	*Acetoin*	*63*			*13*	*20.6*	*50*	*79.4*
	2,3-Pentanedione	*33*			*1*	*3.0*	*32*	*97.0*
	2,3-Butanedione	*54*					*54*	*100*
	Butyl butyrolactate	*21*					*21*	*100*
Herbal	*1-Hexanol*	*42*			*7*	*16.7*	*35*	*83.3*
	Piperitone	*24*			*1*	*4.2*	*23*	*95.8*
	β-Pinene	*59*					*59*	*100*
	α-Pinene	*46*					*46*	*100*
	Eucalyptol	*39*					*39*	*100*
	Linalyl acetate	*20*					*20*	*100*
	1,4-Cineol	*17*					*17*	*100*
	Thymol[$]	*7*					*7*	*100*
	γ-Pentalactone	*5*					*5*	*100*
Balsamic	*Methyl cinnamate*	*54*			*3*	*5.6*	*51*	*94.4*
	Ethyl cinnamate	*19*			*5*	*26.3*	*14*	*73.7*
	Benzyl benzoate	*37*					*37*	*100*
	Cinnamyl alcohol	*10*					*10*	*100*
	Benzophenone[#]	*2*					*2*	*100*
	Benzyl cinnamate[#]	*2*					*2*	*100*
Tonka	*Coumarin*	*21*			*4*	*19.0*	*17*	*81.0*
	Hydrocoumarin	*47*					*47*	*100*
Green	*(3Z)-3-Hexen-1-ol*	*130*			*8*	*6.2*	*122*	*93.8*
	3-Hexen-1-ol, acetate	*61*					*61*	*100*

	Hexyl hexanoate	12			12	100
	Hexyl 2-methylbutyrate[#]	4			4	100
	Styralyl acetate	4			4	100
	Methyl 2-octynate	1			1	100
Citrus	Limonene	95	3	3.2	92	96.8
	Citral	33			33	100
	6-Methyl-5-heptene-2-one	5			5	100
Nutty	2,3,5-trimethylpyrazine	39			39	100
	2,3-dimethylpyrazine	19			19	100
	2,3,5,6-tetramethylpyrazine	17			17	100
	2-ethyl-3-methylpyrazine	9			9	100
	Dimethyl butanedioate	1			1	100
Coconut	δ-Decalactone	107			107	100
	γ-Nonalactone	75			75	100
	γ-Octalactone	64			64	100
	Coumarin, 6-methyl	6			6	100
	γ-Heptalactone	1			1	100
Waxy	Ethyl laurate	11			11	100
	Ethyl decanoate	6			6	100
	Ethyl octanoate	5			5	100
	Ethyl nonanoate	3			3	100
Terpinic	α-Terpineol	98			98	100
	p-Cymene	53			53	100
	γ-Terpinene	39			39	100
Chocolate	2,5-dimethylpyrazine	11			11	100
	Isopentyl phenylacetate	9			9	100
	2-Methoxy-3-methylpyrazine	5			5	100
Fermented	Isopentyl Alcohol	47			47	100
	1-Pentanol	15			15	100
Bready	Furfuryl alcohol	33			33	100
	Furfural*	16			16	100
Woody	α-Caryophyllene	14			14	100
	[c]2-Hy-3,5,5-t-cyclohex-2-en	12			12	100
Anisic	p-Anisaldehyde	50			50	100
	Estragole (4-allyanisole)	15			15	100
Popcorn	Acetylpyrazine	53	1	1.9	52	98.1
Meaty	Hemineurine	44	1	2.3	43	97.7
Smoky	Syringol	7	1	14.3	6	85.7
Phenolic	Guaiacol (2-methoxyphenol)	53			53	100
Tropical	δ-Dodecalactone	53			53	100
Musty	2-Acetylpyrrole	10			10	100
Camphoreous	Fenchol	9			9	100
Earthy	cis-Linalool oxide	6			6	100
Honey	Methyl phenylacetate	2			2	100
Odorless	Caffeine	1			1	100
	Strawberry glycidate_A	23	1	4.3	22	95.7
	Strawberry glycidate_B	12	1	8.3	11	91.7
	Heliotropine PG acetal	35			35	100
	4-methylbenzyl alcohol	3			3	100
	Aromadendrene	3			3	100

Note:
[a] # = frequency of occurrence of flavor chemicals in each concentration category
[b] % = Percentage of occurrence of flavor chemicals in each concentration category
[c]2-Hydroxy-3,5,5-trimethyl-cyclohex-2-en is shortened as 2-Hy-3,5,5-t-cyclohex-2-en

Supplemental Table 2: Total Concentration of Flavor Chemicals in 277 EC Refill Fluids

	Product Code	TFC (mg/ml)
1	U48	362.3
2	U47	118.2
3	U28	68.4
4	U30	67.5
5	U129	64.7
6	U112	63.1
7	U36	54.6
8	U85	54.0
9	U97	53.4
10	U96	52.0
11	U179	50.2
12	U109	49.5
13	U104	48.6
14	N28	47.4
15	U37	47.2
16	C21	47.1
17	U149	46.7
18	N27	46.7
19	C18	44.7
20	U99	43.5
21	U98	42.3
22	U73	40.3
23	U66	39.9
24	U113	38.5
25	U151	37.0
26	U188	35.6
27	U143	33.8
28	U127	33.8
29	U158	33.2
30	N2	32.6
31	U170	32.4
32	U193	31.5
33	U197	30.4
34	U59	30.1
35	U60	29.6
36	U27	29.5
37	U34	28.6
38	U166	28.6
39	U110	28.5
40	B7	27.7
41	U65	27.3
42	U23	27.1
43	U86	27.1
44	U12	26.9
45	C23	26.9
46	U160	26.9
47	B3	26.6
48	U165	26.3
49	U4	25.5
50	U74	25.1
51	U72	24.8
52	N6	23.4
53	N13	23.2
54	N5	22.7
55	U161	22.4
56	N12	21.8
57	U139	21.3
58	U49	20.7
59	U135	20.4
60	U141	19.5
61	U136	19.4
62	U69	19.3
63	U168	18.9
64	U57	18.8
65	U45	18.5
66	U138	18.3
67	U105	18.2
68	U122	18.1
69	U137	17.5
70	U178	17.5
71	U63	17.3
72	U62	17.0
73	U114	16.7
74	N7	17.0
75	U128	16.7
76	U102	15.9
77	U192	15.9
78	U51	15.7
79	U58	15.3
80	U93	15.2
81	U107	15.1
82	U119	14.3
83	U77	14.1
84	C26	13.8
85	U95	13.5
86	N23	13.3
87	U184	13.0
88	N24	13.0
89	U131	12.4
90	U130	12.1
91	N25	12.0
92	U183	12.0
93	U196	11.3
94	U25	11.2
95	U11	10.8
96	B1	10.7
97	U155	10.6
98	U163	10.6
99	U64	10.5
100	U67	10.4
101	U169	10.1
102	U186	10.1
103	U32	9.7
104	U2	9.7
105	U55	9.3
106	C2	9.3
107	U156	9.3
108	U14	9.1
109	U71	9.1
110	U150	8.5
111	U133	8.4
112	U194	8.3
113	U148	7.9
114	U121	7.5
115	U84	7.5
116	U146	7.4
117	U79	7.1
118	U39	6.9
119	U91	6.7
120	U126	6.7
121	U142	6.5
122	U116	6.4
123	U132	6.2
124	U61	6.1
125	U176	5.9
126	U7	5.8
127	C5	5.7
128	U103	5.7
129	U13	5.6
130	N8	5.6
131	U123	5.6
132	U187	5.6
133	U117	5.5

134	U100	5.5
135	U17	5.4
136	B8	5.4
137	U68	5.4
138	U144	5.4
139	N1	5.3
140	N11	5.3
141	U31	5.3
142	U124	5.3
143	N9	5.2
144	U26	5.2
145	N10	5.2
146	U53	5.1
147	U134	5.1
148	U111	5.0
149	C16	4.8
150	U152	4.8
151	U172	4.8
152	U173	4.8
153	U50	4.7
154	U29	4.7
155	U185	4.6
156	U56	4.6
157	U180	4.5
158	U120	4.4
159	U75	4.4
160	U171	4.3
161	U140	4.3
162	U175	4.3
163	N3	4.2
164	U52	4.2
165	U174	4.1
166	U46	4.0
167	U189	3.9
168	N22	3.8
169	U195	3.8
170	U118	3.7
171	U44	3.5
172	U177	3.5
173	U181	3.2
174	U125	3.2
175	U33	3.2
176	U145	3.0
177	U3	2.9
178	U182	2.9
179	N33	2.8
180	U147	2.7
181	N32	2.7
182	N34	2.7
183	U191	2.7
184	U88	2.7
185	U154	2.6
186	N4	2.6
187	U94	2.6
188	C25	2.6
189	N20	2.5
190	U10	2.5
191	U198	2.5
192	N15	2.4
193	N21	2.4
194	N14	2.4
195	C27	2.4
196	U87	2.3
197	C8	2.3
198	U76	2.3
199	U190	2.3
200	N19	2.2
201	N17	2.2
202	U20	2.2
203	N16	2.2
204	N18	2.2
205	U81	2.2
206	U92	2.2
207	U164	2.2
208	U43	2.2
209	U16	2.1
210	U108	2.1
211	N26	2.1
212	B5	2.0
213	C4	2.0
214	C9	1.9
215	C11	1.9
216	U1	1.7
217	U13	1.7
218	B2	1.7
219	C1	1.7
220	U21	1.7
221	C22	1.7
222	U42	1.7
223	U115	1.7
224	U35	1.5
225	B6	1.5
226	C7	1.5
227	U22	1.5
228	U159	1.5
229	U14	1.5
230	N29	1.4
231	U70	1.3
232	C10	1.2
233	U157	1.2
234	U18	1.1
235	C6	1.1
236	U38	1.0
237	U167	1.0
238	N31	1.0
239	U19	1.0
240	U15	1.0
241	N30	0.9
242	N36	0.9
243	N38	0.9
244	N35	0.8
245	N37	0.8
246	U9	0.8
247	U82	0.8
248	C3	0.7
249	C15	0.7
250	U41	0.7
251	U101	0.7
252	C24	0.7
253	B4	0.6
254	U54	0.6
255	C14	0.6
256	U89	0.6
257	U83	0.6
258	U106	0.6
259	U12	0.5
260	U90	0.5
261	U8	0.5
262	U6	0.4
263	C19	0.4
264	U80	0.4
265	U162	0.4
266	U153	0.3
267	U40	0.2
268	C13	0.2
269	U24	0.1
270	C12	0.1
271	C17	0.03
272	U15	0.01
273	U78	0.01
274	C20	0.01
275	U11	0.005
276	U5	0.000
277	U16	0.000

Supplemental Table 3: Brand/Manufacturer and Product Names of All 277 EC Refill Fluid Products

Brand/Company Name	Product Name
Canyon Crest Vape S	Cinnamon Bomb Fiery
Canyon Crest Vape S	Cinnamon Bomb
Flavor Art	Menthol Arctic
Johnson Creek	Tundra
NicQuid	Sinthol
Beard Vape Co	No. 64
LiQua	Two Apples
LiQua	Mints
LiQua	Two Apple
LiQua	Two Apple
Cosmic Fog	The Shocker
Beard Vape Co	No. 51
NJOY Artist Collection	Paramour
LiQua	Two Apples
LiQua	Two Apples
LiQua	Two Apples
The Mad Alchemist	Dragons Breath
LiQua	Ry4 Tob.
LiQua	Ry4 Tob.
LiQua	Ry4 Tob.
LiQua	Ry4 Tobacco
LiQua	Q Honeydew Drop
Cuttwood	Sugar Drizzle
Beard Vape Co	No. 88
The Mad Alchemist	Winters Bite
Twelve Vapor	Libra
NicQuid	Smoothol
Mystique Vapor	Prometheus
Seduce Juice	Snake Eyes
LiQua	Menthol
Cuttwood	Sugar Drizzle
Twelve Vapor	Aries
Glas	Pound Cake
Cuttwood	Boss Reserve
Kilo	Dewberry Cream
LiQua	Q Peach
Kilo	Dewberry Cream
Cuttwood	Boss Reserve
Beard Vape Co	No. 05
LiQua	Q Peach
The Milkman	Churios
LiQua	Peach
LiQua	Two Mints
LiQua	Q Peach
LiQua	Peach
LiQua	Peach
Seduce Juice	Snake Oil
Seduce Juice	Snake Bite
LiQua	Peach
LiQua	Q Pina Colada
Beetle Vapour Juice	Blueberry Hills
LiQua	Menthol
SMKING	Strawberry
LiQua	Menthol
Seduce Juice	Snake Venom
SMKING	Strawberry
Vapor Jerry's	Death Before Dishonor
Canyon Crest Vape S	Dewberry Cream
NicQuid	Sublime
Vapor Jerry's	Oh R'Lyeh
The Milkman	The Milkman
Majestic	King Kong
Cuttwood	Unicorn Milk
Liquid State	Apple Butter
Canyon Crest Vape S	Rainbow Sherbert
Vapor Jerry's	Berry Untraditional
NJOY Artist Collection	Dragonscape
Bombies	Kiss The Ring
The Milkman	Churrios
Cosmic Fog	Cola Gummy
Pop Drops	S'mores vape juice
LiQua	Menthol
Kilo	Kiberry Yoghurt
Bombies	Bacco B
NicQuid	Daybreak
NJOY Artist Collection	Hedon's Bite
Twelve Vapor	Taurus
Canyon Crest Vape S	WTF
Cuttwood	Mega Melons
LiQua	HP Sweet Accelerator
ANML	Looper
Bombies	Agent P
LiQua	Q Menthol

LiQua	Cheesecake
Naked 100	Naked 100
LiQua	Q Menthol
LiQua	Menthol
LiQua	Menthol
Space Jam	Andromeda
NicQuid	Strawnanna Smoothie
NicQuid	Southern Freeze
LiQua	Menthol
Space Jam	Starship 1
Glas	Milk
LiQua	Q Menthol
LiQua	Q Menthol
LiQua	Menthol
The Mad Alchemist	Eye of Newt
Seduce Juice	White Walker
Lost Art LiQuids	Unicorn Puke
Cuttwood	Mega Melons
Space Jam	Eclipse
LiQua	Menthol
Lost Art LiQuids	Unicorn Puke
LiQua	Menthol
Daze	#Crawlie Tuesday
The Mad Alchemist	Custard Matter
LiQua	Menthol
Blue Label Elixir	Famous
The Mad Alchemist	Zen
NicQuid	Maui
Twelve Vapor	Pisces
The Mad Alchemist	Snow White's Demise
LiQua	Energy Drink
Bombies	Nana Cream
Mystique Vapor	Cronos
LiQua	French Pipe Tob
LiQua	Q Cherribakki
LiQua	HP Overdrive
NicQuid	Strawberry Fuzz
Vapor Jerry's	Cut 'N Run
Bombies	White Gummy B
NicQuid	Peach Lemonade

Kilo	Fruit Whip
Cosmic Fog	Church
LiQua	Coffee
LiQua	Coffee
NJOY Artist Collection	Samba Sun
LiQua	Q Apple
LiQua	MB
NicQuid	Banana Nut Bread
Space Jam	Galactica
LiQua	Apple
Bombies	Black Out City
LiQua	Q Apple
LiQua	Coffee
Q Vapor Labs	North Shore
Mystique Vapor	Oceanus
LiQua	MB
Lost Art LiQuids	Gummy Glu
NicQuid	Blueberry
LiQua	MB
LiQua	MB
LiQua	Q Apple
Lion	Love Potion
NicQuid	Midnight Express
Beard Vape Co	No. 71
SMKING	Strawberry
LiQua	Mild Kretek Tob.
The Mad Alchemist	Chem Trail
Cosmic Fog	Lost Fog Streak
Cosmic Fog	Lost Fog Baie Creme
Johnson Creek	Arctic Menthol
Canyon Crest Vape S	Ho!Ho! Watermelon
Space Jam	Pluto
O.M.G E LiQuid	WTF
Cosmic Fog	Nutz
LiQua	Q Double Bubble
Bombies	Tiger Style
Vapor Jerry's	Jugo De Las Muerta
Cosmic Fog	Euphoria
Cosmic Fog	Kryptonite
LiQua	Bright Tobacco
Daze	#Selfie Sunday

Cosmic Fog	Lost Fog Neon Cream
Canyon Crest Vape S	Melon Mania
Twelve Vapor	Scorpio
LiQua	Bright Tobacco
Twelve Vapor	Aquarius
Bombies	Seven Seas
LiQua	Q Blackberry Jack
Cosmic Fog	Milk and Honey
NicQuid	Soho
Space Jam	Pulsar
Opulence Elixir	Popsuckle
Mystique Vapor	Hyperion
LiQua	Apple
Space Jam	Astro
LiQua	MB
LiQua	MB
LiQua	MB
LiQua	Watermelon
Mystique Vapor	Asteria
Twelve Vapor	Gemini
Thrive	Ice Lemon Cole
LiQua	Brownie
LiQua	HP Summer Drift
The Mad Alchemist	Twice in a Blue Moon
LiQua	Licorice
LiQua	Watermelon
Glas	Pebbles
Thrive	Ice Lemon Cole
Thrive	Ice Lemon Cole
LiQua	Watermelon
LiQua	Licorice
LiQua	Tiramisu
LiQua	Q Fragola Fresca
LiQua	Watermelon
Twelve Vapor	Cancer
Thrive	Banana
Thrive	Banana
Thrive	Banana
LiQua	Tiramisu
LiQua	Watermelon
LiQua	Banana

LiQua	HP Fruity Velocity
Seduce Juice	Jezebel
LiQua	Apple
LiQua	Strawberry
LiQua	Q The Moment
ANML	Carnage
LiQua	Apple
LiQua	Apple
LiQua	Cherry
LiQua	Bright Tob.
LiQua	Bright Tob.
LiQua	Bright Tob.
LiQua	Bright Tob.
LiQua	Bright Tob.
LiQua	Cherry
LiQua	Berry Mix
LiQua	Q Piedmont Sunrise
Bombies	Product X
LiQua	Q Berry Mix
LiQua	Q Berry Mix
LiQua	Grape
LiQua	Strawberry
Ripe Vapes	RF- HSAC
Seduce Juice	Blackjack
LiQua	Vanilla
Seduce Juice	Exodus 7:20
LiQua	Strawberry
Vape Mail	Overnight
LiQua	Berry Mix
LiQua	Grape
LiQua	Vanilla
LiQua	Citrus Mix
LiQua	Citrus Mix
LiQua	Citrus Mix
Cuttwood	Bird Brains
LiQua	Vanilla
LiQua	Vanilla
LiQua	Vanilla
LiQua	Berry Mix
LiQua	Vanilla
LiQua	Vanilla
LiQua	Vanilla
LiQua	Berry Mix

LiQua	Virginia Tobacco
LiQua	Q Golden Roanoke Tobacco
LiQua	Cappucino
LiQua	Berry Mix
LiQua	Red Oriental Tobacco
LiQua	Cola
Cuttwood	Bird Brains
LiQua	Red Oriental Tobacco
LiQua	Chocolate
NJOY Artist Collection	Sacre Coeur
LiQua	Cola
LiQua	Q Turkish Tobacco
LiQua	Chocolate
LiQua	Turkish Tobacco

LiQua	Vermillion Oriental Tob.
LiQua	Blueberry
Seduce Juice	Caesar
The Mad Alchemist	Number 9
LiQua	Goldenrod Oriental Tob.
LiQua	Q Havana Libre Tob.
LiQua	Cuban Cigar Tobacco
LiQua	Golden Oriental Tob.
LiQua	Traditional Tobacco
LiQua	Q Traditional Tobacco
LiQua	Q Traditional Tobacco
LiQua	Traditional Tobacco
LiQua	American Blend Tob.
LiQua	Q American Blend Tob.
LiQua	American Blend Tob.

Supplemental Figure 1: Frequency Distribution of 72 Flavor Chemicals

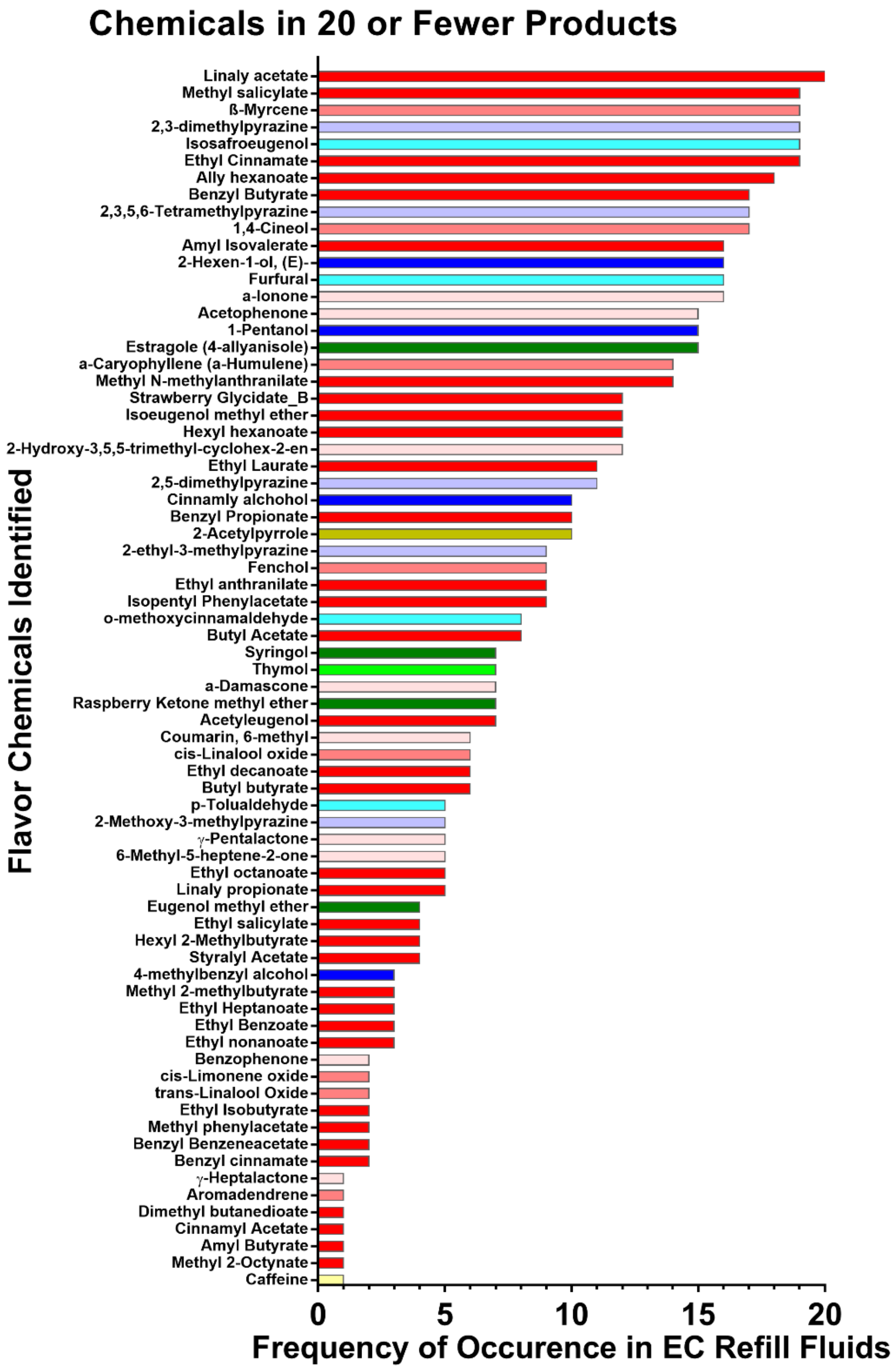

Supplemental Figure 1: Frequency Distribution of 72 Flavor Chemicals. (A) shows 72 out of 155 flavor chemicals that occurred 20 or fewer times in at least one product. The *x*-axis is the number of refill fluids in which the chemicals were found and the *y*-axis is sorted according to decreasing frequency of their occurrence. Frequency ranged from 1 – 20 with the highest being linalyl acetate to the lowest which occurred only once (δ-heptalactone, aromadendrene, dimethyl butanedioate, cinnamyl acetate, amyl butyrate, methyl 2-octynate and caffeine).
